# Safety and tolerability of intravenous immunoglobulin in patients with active dermatomyositis: results from the randomised, placebo-controlled ProDERM study

**DOI:** 10.1186/s13075-023-03232-2

**Published:** 2024-01-17

**Authors:** Rohit Aggarwal, Joachim Schessl, Christina Charles-Schoeman, Zsuzsanna Bata-Csörgő, Mazen M. Dimachkie, Zoltan Griger, Sergey Moiseev, Chester V. Oddis, Elena Schiopu, Jiri Vencovský, Irene Beckmann, Elisabeth Clodi, Todd Levine, the ProDERM investigators

**Affiliations:** 1 Division of Rheumatology and Clinical Immunology, University of Pittsburgh School of Medicine, Pittsburgh, PA USA; 2 Department of Neurology, Friedrich-Baur-Institute, Ludwig-Maximilians University of Munich, Munich, Germany; 3 University of California, Los Angeles, CA USA; 4 Department of Dermatology and Allergology, University of Szeged, Szeged, Hungary; 5 Department of Neurology, University of Kansas Medical Center, Kansas City, KS USA; 6 Division of Clinical Immunology, Faculty of Medicine, University of Debrecen, Debrecen, Hungary; 7 Tareev Clinic of Internal Diseases, Sechenov First Moscow State Medical University, Moscow, Russia; 8 Division of Rheumatology, Medical College of Georgia at Augusta University, Augusta, GA USA; 9 Institute of Rheumatology and Department of Rheumatology, 1st Medical Faculty, Charles University, Prague, Czech Republic; 10 Octapharma Pharmazeutika Produktionsges. m.b.H., Vienna, Austria; 11 Phoenix Neurological Associates, Ltd, Phoenix, AZ USA

**Keywords:** Dermatomyositis, Intravenous immunoglobulin, Myositis, Safety, Tolerability

## Abstract

**Background:**

Dermatomyositis is an idiopathic inflammatory myopathy characterised by rashes and progressive muscle weakness. The recent ProDERM (Progress in DERMatomyositis) study is the first large randomised, placebo-controlled trial to establish the efficacy and safety of intravenous immunoglobulin (IVIg) in adult patients with dermatomyositis. Objectives of this analysis were to closely examine the safety and tolerability of IVIg in patients from the ProDERM study.

**Methods:**

ProDERM was a double-blind, randomised, placebo-controlled, multicentre, phase 3 study. In the first period (weeks 0–16), adults with active dermatomyositis received 2.0 g/kg IVIg (Octagam 10%; Octapharma AG) or placebo every 4 weeks. In the open-label extension period (weeks 16–40), all patients received IVIg for 6 additional cycles; dose reduction (1.0 g/kg) was permitted if patients were stable. Treatment-emergent adverse events (TEAEs) were documented.

**Results:**

The 95 patients enrolled were randomised to receive IVIg (*N* = 47) or placebo (*N* = 48) in the first period, with 5 switching from placebo to IVIg. Overall, 664 IVIg infusion cycles were administered. During the first period, 113 TEAEs were possibly/probably related to treatment in 30/52 patients (57.7%) receiving IVIg and 38 in 11 patients (22.9%) on placebo. Eight patients discontinued therapy due to IVIg-related TEAEs. Eight thromboembolic events (TEEs) occurred in six patients on IVIg; six in five patients were deemed possibly/probably related to IVIg. Patients with TEEs exhibited more baseline TEE risk factors than those without TEEs (2.4–15.2-fold higher). Lowering infusion rate reduced the rate of TEEs, and none occurred at the lower IVIg dose. No haemolytic transfusion reactions or deaths occurred.

**Conclusions:**

Results from this study demonstrate that IVIg has a favourable safety profile for treatment of adult dermatomyositis patients and provides evidence that will help to inform treatment choice for these patients. Dermatomyositis patients receiving high-dose IVIg should be monitored for TEEs, and a low rate of infusion should be used to minimise TEE risk, particularly in those with pre-existing risk factors.

**Trial registration:**

ProDERM study (NCT02728752).

**Supplementary Information:**

The online version contains supplementary material available at 10.1186/s13075-023-03232-2.

## Background

Dermatomyositis is an idiopathic inflammatory myopathy characterised by rashes and progressive muscle weakness [1]. It is estimated to affect between 1 and 13 people per 100,000 of the US population [2, 3]. Although the pathogenesis of dermatomyositis is unknown, several genetic, immunologic and environmental factors have been implicated [4].

Glucocorticoids and other immunosuppressive drugs are widely used in the treatment of dermatomyositis but are often associated with significant adverse effects. In addition, patients with myositis have a high rate of mortality due to infections [5, 6]. Intravenous immunoglobulins (IVIg) are highly purified immunoglobulin G concentrates prepared from human plasma and are widely used in the treatment of autoimmune and inflammatory disorders [7, 8]. IVIg is recommended in European guidelines as a glucocorticoid-sparing agent and is used off-label for dermatomyositis, usually in combination with immunosuppressive drugs [9–11]. However, there has been a lack of large, randomised studies to support the use of IVIg in this patient population.

The ProDERM (Progress in DERMatomyositis) study recently established the efficacy, safety and tolerability of IVIg in adult dermatomyositis patients in a large, randomised, placebo-controlled trial [12, 13]. The study showed that significantly more patients responded to IVIg than placebo (78.7% versus 43.8%, respectively). The results of the ProDERM study led to the approval of IVIg (Octagam 10%) for treatment of dermatomyositis in the USA, Canada and most European countries [14–16].

Here, we present detailed analyses of the safety and tolerability of IVIg in patients with dermatomyositis from the ProDERM study.

## Methods

### Study design

Details of the ProDERM study (NCT02728752) protocol have been published previously [13]. In summary, the study was a prospective, double-blind, randomised, parallel-group, placebo-controlled, multicentre, phase 3 study including dermatomyositis patients from 36 European and North American centres. Enrolment started in February 2017, and the last patient visit was in November 2019. Aims of this analysis were to closely examine the safety and tolerability of IVIg in patients from ProDERM. The study was conducted in accordance with the Declaration of Helsinki, in compliance with good clinical practice guidelines, and was approved by the relevant independent ethics committees or institutional review boards, as applicable. Informed consent was obtained from each patient before any study-related procedures were conducted.

### Patients

Full inclusion and exclusion criteria have been described previously [13]. In summary, patients aged ≥ 18 and < 80 years with muscle weakness and definite or probable active dermatomyositis according to the Bohan and Peter criteria [17, 18], as determined by an adjudication committee, were eligible for inclusion. Patients with any history of thromboembolic events (TEEs) such as deep vein thrombosis (DVT), pulmonary embolism (PE), myocardial infarction, ischemic stroke, transient ischemic attack or peripheral artery disease (Fontaine IV) were excluded, as were patients with known blood hyperviscosity, or other hypercoagulable states [13].

### Study procedures

**Fig. 1:**
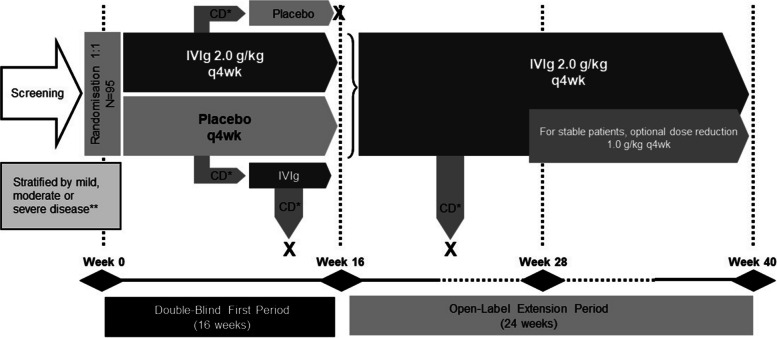
Study design. X, drop-out. *CD, confirmed deterioration. Defined as change from baseline on two consecutive visits in Physician’s Global Disease Activity VAS worsening ≥ 2 cm and MMT-8 worsening ≥ 20%, OR global extra-muscular activity worsening ≥ 2 cm on the MDAAT VAS, OR any three of five CSM (core set measures, excluding enzymes) worsening by ≥ 30%). **Physician’s Global Disease Activity (GDA) value of 0–3 (mild), 4–6 (moderate), 7–10 (major). #Placebo patients having confirmed deterioration at week 16 continued in open-label part

In the double-blind first period (weeks 0–16), eligible patients were randomised 1:1 to receive up to four infusion cycles of either 2.0 g/kg IVIg (Octagam 10%; Octapharma AG, Lachen, Switzerland) or placebo every 4 weeks (Fig. 1). Infusions were given on two consecutive days, and the infusion cycle could be prolonged up to 5 days, based on tolerability, at the discretion of the investigator. Each infusion cycle included all infusion episodes administered over the 2- to 5-day visit. Patients who had confirmed deterioration, as defined by Aggarwal et al. (2021) [13, 19] in the first period, crossed over to the alternative treatment at week 8 or week 12.

The open-label extension period (weeks 16–40) included all patients except those who had confirmed deterioration while on IVIg. In this period, all patients received 2.0 g/kg IVIg every 4 weeks for a further 6 infusion cycles. An IVIg dose reduction to 1.0 g/kg was permitted from week 28 for patients who were stable. The overall period included both the first and extension periods. Full details of the study procedures have been described previously [13].

### Concomitant medications and premedications

At study entry, the maximum permitted glucocorticoid dose was 20 mg daily prednisone equivalent, with initial doses maintained during the first period. Other immunosuppressive drugs were permitted in stable doses throughout the first period, but additional immunosuppressive rescue medication was not permitted during the study. Opioids and nonsteroidal anti-inflammatory drugs were permitted if the treatment regimen was stable from 2 weeks prior to enrolment until the end of the first period. Dose reduction of concomitant dermatomyositis medication was permitted in the extension period at the discretion of the investigator [13]. Premedication to alleviate side effects could only be administered for patients who experienced infusion-related adverse events (AEs) at two previous consecutive visits that were considered likely to be prevented by mild analgesics, antihistamines, antipyretics or antiemetic drugs.

Prophylaxis for TEEs was permitted where deemed necessary by the investigator as a precautionary measure and followed standard of care.

### Safety assessment

Treatment-emergent AEs (TEAEs) were defined as those that occurred during the first or extension periods, following administration of the first or subsequent doses of study drug. TEAEs and serious TEAEs, with particular emphasis on TEAEs of special interest, i.e. TEEs and haemolytic transfusion reactions, along with fatalities, were documented throughout the study and up to 4 weeks after the last administration of IVIg or placebo. TEEs, including DVT and PE, were assessed at each visit using the Wells criteria [20], modified according to NICE clinical guideline 144, 2012 [21].

TEAEs were considered to be associated with the most recent treatment administered. TEAEs were classified as ‘infusional’ if the onset was during the infusion cycle or within 72 h after the end of the last infusion episode of the respective infusion cycle/visit.

All TEAEs were rated by the blinded local site investigator as nonserious or serious, as per standard definition, with serious defined as any TEAE that resulted in death, was life-threatening, required hospitalisation or prolongation of existing hospitalisation, resulted in persistent or significant disability/incapacity or was another important medical event, including TEEs. Wells scores were recorded to assess the probability of DVT or PE. TEAEs were also rated by the blinded investigator for severity, with mild TEAEs being usually transient, which caused discomfort but did not interfere with the patient’s routine activities, moderate TEAEs being sufficiently discomforting to interfere with the patient’s routine activities, and severe TEAEs being incapacitating and preventing the pursuit of the patient’s routine activities. The relationship of TEAEs to the administered IVIg or placebo was assessed by the investigator.

An independent data monitoring committee was set up to independently review safety data, to review TEEs and monitor the stopping rules and to give advice on the continuation, modification or termination of the study.

### Statistical methods

Statistical methods are as described previously [12, 13]. Safety analyses were performed on the safety analysis set, which included all subjects who received at least part of one infusion of IVIg or placebo. Whereas general baseline information was summarised by randomised treatment, AE data was summarised in tables according to the most recent treatment administered, IVIg or placebo. Patients who switched to the other treatment during the first study period are therefore considered to be at risk for AEs in both treatment groups. The safety analysis comprised descriptive statistics, tabulations and listings of all AEs and other safety-relevant endpoints.

For analyses and reporting purposes, AEs were coded with MedDRA (version 18.1) and medications with the WHODrug Dictionary (version Sep 2015).

## Results

### Patient demographics and baseline characteristics

Of 126 patients screened, 95 were enrolled in the study, with 47 randomised in the first period to receive IVIg and 48 randomised to receive placebo. All enrolled patients received at least one infusion of study drug and were thus included in the safety analysis set and analysed according to the intention-to-treat principle. Of patients randomised to receive IVIg, 45 (95.7%) completed the first period, as did 46 (95.8%) in the placebo group. Five patients (10.4%) on placebo crossed over to IVIg during the first period, with no patients on IVIg switching to placebo. A total of 69 (72.6%) patients completed the extension period. Full details of patient disposition were described by Aggarwal et al. (2022) [12].

Demographics and baseline characteristics were generally balanced between groups [12]. Briefly, the median (range) age was 52.0 years (22.0–79.0), and 71 (74.7%) patients were female. Median time since diagnosis was 2.6 years (0.1–48.7). All patients exhibited symmetric proximal muscle weakness and typical skin rash, with a mean MMT-8 score of 120.9 (maximum 150), and 67 patients (70.5%) had dermatomyositis classed as ‘definite’. The use of concomitant therapy was similar between the two treatment groups, with glucocorticoids taken by 88.4% of patients and non-glucocorticoid medications taken by 68.4%.

During the study, 664 infusion cycles were administered, with a median dose of 2.0 g/kg IVIg. The median duration of infusion cycles was 2.4 days, with 76 (80.0%) patients receiving IVIg over ≤ 2 days. A total of 33 (34.7%) patients had IVIg infusion cycles over 3 days, 12 (12.6%) patients received IVIg over 4 days and 2 (2.1%) patients received IVIg infusions over 5 days (some patients received IVIg over more than one duration).

### Overview of adverse events

A summary of adverse events experienced during the study is presented in Table 1. All adverse events were deemed to be TEAEs.

**Table 1:** Overview of adverse events

Category of TEAE and intensity	First period				Overall period	
	**IVIg (*n* = 52**^a^**)**		**Placebo (*n* = 48)**		**All IVIg (*n* = 95)**	
	**No. of pts (%)**	**No. of events**	**No. of pts (%)**	**No. of events**	**No. of pts (%)**	**No. of events**
TEAEs	42 (80.8%)	196	28 (58.3%)	135	84 (88.4%)	545
Infusional TEAEs	34 (65.4%)	139	19 (39.6%)	65	76 (80.0%)	351
Serious TEAEs	3 (5.8%)	5	2 (4.2%)	4	14 (14.7%)	22
TEAEs related to study drug	30 (57.7%)	113	11 (22.9%)	38	62 (65.3%)	282
TEAEs leading to discontinuation of study drug	3 (5.8%)	8	0 (0.0%)	0	13 (13.7%)	25
TEEs	1 (1.9%)	2	0 (0.0%)	0	6 (6.3%)	8
Related^b^ TEEs	0 (0.0%)	0	0 (0.0%)	0	5 (5.3%)	6
HTRs	0 (0.0%)	0	0 (0.0%)	0	0 (0.0%)	0
Deaths	0 (0.0%)	0	0 (0.0%)	0	0 (0.0%)	0

*HTR* haemolytic transfusion reaction, *IVIg* intravenous immunoglobulin, *N* number of patients, *n* number of events, *TEAE* treatment-emergent adverse event, *TEE* thromboembolic event

^a^Includes five patients that switched from placebo to IVIg during the first period

^b^Includes TEAEs deemed possibly or probably related to the infusion by the investigator

During the first period, 42 patients (80.8%) who received IVIg experienced a total of 196 TEAEs and 28 patients (58.3%) who received placebo experienced 135 TEAEs (Table 1). Of these, there were 113 treatment-related TEAEs in 30 patients (57.7%) in the IVIg group and 38 related TEAEs in 11 patients (22.9%) in the placebo group.

In the overall period, 84 patients (88.4%) experienced 545 TEAEs following treatment with IVIg (Table 1). Of these, 282 TEAEs in 62 patients (65.3%) were assessed as related to the study drug (Suppl. Table 1). Most of these related TEAEs (260/282; 92.20%) occurred during or within 72 h of an infusion cycle and were classed as infusional TEAEs, whereas in the placebo group, 29/38 (76.32%) were classed as infusional TEAEs. The most commonly reported IVIg-related TEAEs (> 5% of patients) were headache (42%), fever (19%), nausea (16%), vomiting (8%), chills (7%), musculoskeletal pain (7%) and increased blood pressure (6%) (Suppl. Table 1). Of the patients who received infusions over ≤ 2 days, 42 (54.6%) experienced a related TEAE compared with 27 patients (71.1%) who received infusions over > 2 days (most likely due to patients having their infusion cycles lengthened due to such side effects).

### Adverse events stratified by intensity and seriousness

Most TEAEs experienced during the study were deemed related to the study drug and were mild in intensity. In the first period, for patients who received IVIg, 82 of 113 related TEAEs were mild, 28 were moderate and 3 were classed as severe (Table 2). In patients who received placebo, 24 mild, 14 moderate, and no severe related TEAEs occurred. The pattern of TEAE intensity with IVIg was similar in the overall period to that seen in the first period; for the overall period, 207 of 282 related TEAEs were classed as mild in intensity, 66 were classed as moderate and 9 were classed as severe. The nine TEAEs of severe intensity were experienced by a total of five patients and included four events of headache and one event each of nausea, muscle spasms, dyspnoea, DVT and PE.

**Table 2:** TEAEs by intensity in the first period and overall period

Category of TEAE and intensity	First period				Overall period	
	**IVIg (*n* = 52**^a^**)**		**Placebo (*N* = 48)**		**All IVIg (*N* = 95)**	
	**No. of pts (%)**	**No. of events (%)**	**No. of pts (%)**	**No. of events (%)**	**No. of pts (%)**	**No. of events (%)**
All TEAEs						
Mild	39 (75.0%)	142 (72.4%)	28 (58.3%)	102 (75.6%)	79 (83.2%)	405 (74.3%)
Moderate	20 (38.5%)	48 (24.5%)	10 (20.8%)	33 (24.4%)	39 (41.1%)	118 (21.7%)
Severe	4 (7.7%)	6 (3.1%)	0 (0.0%)	0 (0.0%)	10 (10.5%)	22 (4.0%)
TEAEs related^b^ to study drug						
Mild	28 (53.8%)	82 (72.6%)	9 (18.8%)	24 (63.2%)	55 (57.9%)	207 (73.4%)
Moderate	13 (25.0%)	28 (24.8%)	5 (10.4%)	14 (36.8%)	25 (26.3%)	66 (23.4%)
Severe	2 (3.8%)	3 (2.7%)	0 (0.0%)	0 (0.0%)	5 (5.3%)	9 (3.2%)

*IVIg* intravenous immunoglobulin, *N* number of patients, *n* number of events, *TEAE* treatment-emergent adverse event

^a^Includes five patients that switched from placebo to IVIg during the first period

^b^Includes TEAEs deemed possibly or probably related to the infusion by the investigator

The latency time and duration for the related TEAEs of headache, nausea, vomiting and fever during the first period are presented in Table 3. In patients who received IVIg, both median latency times and durations for each of the related TEAEs were rather short, ranging from 0 to 3 days, and generally, the latency times and durations of these TEAEs were similar between the IVIg and placebo groups. Latency time and duration of the TEAEs did not appear to change with severity of the TEAE.

**Table 3:** Latency and duration of the related TEAEs of headache, nausea, vomiting and fever stratified by severity (first period)

**TEAE**	**Latency (days)**^a^				**Duration (days)**^b^			
	**IVIg** **(*n* = 52)**		**Placebo** **(*n* = 48)**		**IVIg** **(*n* = 52)**		**Placebo** **(*n* = 48)**	
	*N*	Median (range)	*N*	Median (range)	*N*	Median (range)	*N*	Median (range)
***Headache***								
Mild	32	0.5 (0.0–4.0)	3	0.0 (0.0–14.0)	32	1.0 (1.0–5.0)	2	8.0 (1.0–15.0)
Moderate	12	2.0 (0.0–4.0)	4	0.0 (0.0–0.0)	12	2.5 (1.0–11.0)	4	1.0 (1.0–2.0)
Severe	1	2.1 (2.1–2.1)	0	-	1	3.0 (3.0–3.0)	0	-
All	45	1.0 (0.0–4.0)	7	0.0 (0.0–14.0)	45	1.0 (1.0–11.0)	6	1.0 (1.0–15.0)
***Nausea***								
Mild	8	1.8 (0.0–4.0)	1	3.0 (3.0–3.0)	8	2.5 (1.0–4.0)	1	24.0 (24.0–24.0)
Moderate	3	0.0 (0.0–3.0)	1	2.0 (2.0–2.0)	3	2.0 (1.0–4.0)	1	8.0 (8.0–8.0)
Severe	0	-	0	-	0	-	0	-
All	11	1.6 (0.0–4.0)	2	2.5 (2.0–3.0)	11	2.0 (1.0–4.0)	2	16.0 (8.0–24.0)
***Vomiting***								
Mild	1	2.0 (2.0–2.0)	0	-	1	1.0 (1.0–1.0)	0	-
Moderate	2	1.5 (0.0–3.0)	0	-	2	2.0 (2.0–2.0)	0	-
Severe	0	-	0	-	0	-	0	-
All	3	2.0 (0.0–3.0)	0	-	3	2.0 (1.0–2.0)	0	-
***Fever***								
Mild	14	1.2 (0.0–3.0)	3	0.0 (0.0–1.4)	14	2.0 (1.0–3.0)	3	2.0 (1.0–5.0)
Moderate	1	3.0 (3.0–3.0)	0	-	1	3.0 (3.0–3.0)	0	-
Severe	0	-	0	-	0	-	0	-
All	15	1.3 (0.0–3.0)	3	0.0 (0.0–1.4)	15	2.0 (1.0–3.0)	3	2.0 (1.0–5.0)

*IVIg* intravenous immunoglobulin, *TEAE* treatment-emergent adverse event

^a^Latency time is marked as 0 if the TEAE occurred during infusion (including between infusion episodes of the same cycle), otherwise calculated as days since the start of the infusion cycle

^b^Calculated as (date of resolution - date of onset) + 1

The incidence of serious TEAEs regardless of relationship to the study drug was similar in the two treatment groups during the first period: 3 patients (5.8%) on IVIg experienced 5 serious TEAEs, and 2 patients (4.2%) on placebo experienced 4 serious TEAEs. In the overall period, 7 patients (7.4%) experienced a total of 9 serious TEAEs that were considered related to study drug, as shown in Table 4. Following these related serious TEAEs, 2/7 patients (28.6%) were able to resume treatment with IVIg (loss of consciousness in one case and hypoesthesia [TEE] in another). In another case (cerebral infarction [TEE]), the serious TEAE occurred 12 days after the last infusion of IVIg (Table 4). The median (range) latency time for serious related TEAEs where the last infusion was IVIg was 1.95 days (0.0–29.0), and the median duration of the serious related TEAEs was 14.0 days (1.0–109.0).

**Table 4:** Serious TEAEs assessed as at least possibly related to study drug^a^ (overall period)

Treatment at time of event	Patient	MedDRA preferred term	Intensity	Criteria	Causality	Duration (days)	Outcome	Subsequent study treatment
First period IVIg	Patient 1	Muscle spasms	Severe	Life threatening	Probable	1	Recovered/resolved	Study drug withdrawn
		Dyspnoea	Severe	Life threatening	Probable	1	Recovered/resolved	
Extension period (all IVIg)	Patient 2	Deep vein thrombosis (TEE)	Severe	Hospitalisation and life threatening	Probable	73	Recovered/resolved	Study drug withdrawn
		Pulmonary embolism (TEE)	Severe	Hospitalisation and life threatening	Probable	73	Recovered/resolved	
	Patient 3	Cerebrovascular accident (TEE)	Moderate	Hospitalisation and medically important	Possible	109	Recovered/resolved with sequelae	Study drug withdrawn
	Patient 4	Pulmonary embolism (TEE)	Moderate	Hospitalisation and medically important	Possible	14	Recovered/resolved with sequelae	Study drug withdrawn
	Patient 5	Loss of consciousness	Moderate	Hospitalisation	Probable	1	Recovered/resolved	Dose of study drug unchanged The patient subsequently received a further five cycles of IVIg treatment and did not experience any further serious TEAEs
	Patient 6	Cerebral infarction (TEE)	Moderate	Hospitalisation and medically important	Possible	99	Recovered/resolved with sequelae	The serious TEAE occurred 12 days after the last infusion cycle of IVIg
Extension period (all IVIg)	Patient 7	Hypoesthesia (TEE)	Mild	Medically important	Possible	1	Recovered/resolved	Dose of study drug unchanged The patient subsequently received one further cycle of IVIg treatment

*IVIg* intravenous immunoglobulin, *MedDRA* Medical dictionary for regulatory activities, *TEE* thromboembolic event

^a^Includes TEAEs deemed possibly or probably related to the study drug by the investigator

Serious TEAEs assessed as unlikely related or not related to study drug included sepsis (*n* = 1), PE (*n* = 1), ventricular extrasystoles (*n* = 1), tropical spastic paraparesis (*n* = 1), sinus tachycardia (*n* = 1; 2 events) and hypertension (*n* = 1) in the first period and squamous cell carcinoma (*n* = 1), condition aggravated (*n* = 2), atypical pneumonia (*n* = 1), pneumonia (*n* = 1), cardiac failure congestive (*n* = 1), sepsis (*n* = 1), acute respiratory failure (*n* = 1), acute kidney injury (*n* = 1) and *Escherichia bacteraemia* (*n* = 1) in the extension period.

### Adverse events of special interest

During the overall period, 8 TEEs were documented in 6 patients treated with IVIg (*n* = 664 infusion cycles), and none was reported in patients treated with placebo (*n* = 184 infusion cycles). Of the TEEs, six in five patients were assessed as possibly or probably related to the study drug. The median (range) time to TEE occurrence from the start of the first IVIg infusion was 167 days (142–267) and from the last IVIg infusion prior to the event was 12 days (2–29). Overall, most patients had a Wells score of 0 at their last visit prior to the event, including all patients who experienced TEEs. Characteristics of patients with TEEs and details of their TEE risk factors are presented in Table 5. Four of the five patients who experienced possibly or probably related TEEs had hypertension prior to the study. Other risk factors for TEEs included dyslipidaemia and obesity (both *n* = 2) and hypercholesterolaemia, chronic heart failure, ex-smoker, palpitations, myocardial ischaemia, ventricular dilatation and left atrial dilatation, supraventricular arrhythmia and osteoporosis and fractures of the spine (all *n* = 1). In total, the 89 patients who did not experience TEEs exhibited a total of 51 risk factors from the above-mentioned categories (i.e. an average of 0.6 per patient), versus 16 risk factors among 6 patients who did experience TEEs (i.e. an average of 2.7 per patient), equating to a 4.6-fold difference in the number of risk factors. Compared to patients who did not experience TEEs, patients who experienced TEEs had a higher median age (69.0 versus 51.6 years, respectively) and a numerically higher percentage of occurrence for each of the risk factors analysed, ranging from 2.4- to 15.2-fold higher (Table 6). The six patients with TEEs together experienced a total of 24 related TEAEs (mean, 4 per patient), which was similar to the mean number for all patients (3 per patient). Risk of TEE was highest in patients with three or more risk factors. Global disease activity, disease duration and dosing were similar between groups. The occurrence of these TEEs led to a study protocol amendment, whereby the maximum permitted infusion rate was reduced from 0.12 to 0.04 mL/kg/min. This resulted in a reduction in the incidence of TEEs from 1.54 (95% *CI*: 0.42, 3.94) per 100 patient months to 0.54 (95% *CI*: 0.07, 1.95) following implementation.

**Table 5:** Characteristics of patients with TEEs that were deemed possibly or probably related to study drug

Patient	TEE	Age Sex Race BMI	Total dose Max. infusion speed No. of infusion episodes Active infusion time	Time from start of last IVIg infusion to diagnosis of TEE (days)	Global disease activity	Risk factors for TEEs
Patient 2	DVT; severe	67 years Male White race 26.0 kg/m^2^	180 g; 1.978 g/kg 0.12 mL/kg/min Two infusion episodes infused over 340 min	2	Moderate	• Hypertension • Chronic heart failure • Dyslipidaemia • Ex-smoker
	PE; severe			2		
Patient 3	Cerebrovascular accident (TEE)	79 years Female White race 28.0 kg/m^2^	160 g; 2.025 g/kg 0.08 mL/kg/min Four infusion episodes; infused over 555 min	29	Moderate	• Hypertension • Palpitations • Myocardial ischaemia • Dyslipidaemia
Patient 4	PE; moderate	62 years Male White race 28.7 kg/m^2^	180 g; 1.978 g/kg 0.04 mL/kg/min^a^ Two infusion episodes infused over 590 min	24	Moderate	• Hypertension • Ventricular dilatation and left atrial dilatation • Obesity
Patient 6	Cerebral infarction; moderate	70 years Female White race 22.5 kg/m^2^	110 g; 2.000 g/kg 0.12 mL/kg/min Three infusion episodes infused over 321 min	14	Mild	• Hypertension
Patient 7	Hypoaesthesia; mild	67 years Female White race 35.3 kg/m^2^	170 g; 2.000 g/kg 0.04 mL/kg/min Two infusion episodes infused over 592 min	10	Severe	• Supraventricular arrhythmia • Hypercholesterolaemia • Osteoporosis of the lumbar spine and fractures of the thoracic and lumbar spine • Obesity

*BMI* body mass index, *DVT* deep vein thrombosis, *PE* pulmonary embolism, *TEE* thromboembolic event

^a^Patient 4 received IVIg at a maximum infusion rate of 0.12 mL/kg/min during the first period and at the reduced rate of 0.04 mL/kg/min during the extension period (from week 16, onwards). The PE occurred during the extension period, after the dose had been lowered

**Table 6:** Risk factors for TEEs in patients who did not present with TEEs versus those who presented with TEEs (overall period)

	**No. of TEE (*N* = 89)**	**TEE (*N* = 6)**
Age, mean (range), years	51.6 (22–77)	69.0 (62–79)
Gender, *n* (%) female	68 (76.4)	3 (50)
Race, *n* (%)		
Asian	2 (2.2)	0 (0)
Black or African American	5 (5.6)	0 (0)
White	81 (91.0)	6 (100)
Other	1 (1.1)	0 (0)
TEE risk factors		
Hypertension, *n* (%)	31 (34.8)	5 (83.3)
Chronic heart failure, *n* (%)	1 (1.1)	1 (16.7)
Myocardial ischaemia, *n* (%)	2 (2.2)	1 (16.7)
Dyslipidaemia, *n* (%)	4 (4.5)	2 (33.3)
Dilation ventricular and left atrial dilation, *n* (%)	1 (1.1)	1 (16.7)
Obesity, *n* (%)	3 (3.4)	2 (33.3)
Supraventricular arrhythmia, *n* (%)	0 (0.0)	1 (16.7)
Hypercholesterolaemia, *n* (%)	8 (9.0)	2 (33.3)
Osteoporotic fracture, *n* (%)	1 (1.1)	1 (16.7)
No. of TEE risk factors per patient		
0 risk factors	50 (56.2)	0 (0)
1 risk factor	28 (31.5)	1 (16.7)
2 risk factors	10 (11.2)	0 (0)
3 risk factors	1 (1.1)	5 (83.3)
Physician global disease activity (actual), *n* (%)		
Mild	24 (27.0)	2 (33.3)
Moderate	52 (58.4)	4 (66.7)
Severe	13 (14.6)	0 (0.0)
Maximum infusion rate per patient, median (range), mL/kg/min		
Pre-introduction of lower infusion rate	0.12 (0.04–0.12)	0.12 (0.04–0.12)
Following introduction of lower infusion rate	0.04 (0.02–0.08)	0.04 (0.02–0.08)
Actual IVIg dose, median (range), g/kg	1.99 (0.24–2.03)	1.99 (1.98–2.03)

*IVIg* intravenous immunoglobulin, *TEE* thromboembolic event

No patient experienced a haemolytic transfusion reaction during the study.

### Effect of dose reduction

Of 91 patients who entered the extension period, 8 patients (8.8%) had their IVIg dose reduced from 2.0 to 1.0 g/kg at 28 weeks or thereafter, undergoing a total of 23 infusion cycles at the reduced dose. Two of these patients never experienced any related TEAEs under IVIg treatment. Four patients experienced mild related and expected TEAEs under 2 g/kg dosing but none when treated with reduced dose. One patient experienced several related TEAEs of different severity under placebo, as well as severe headache occurring twice under 2 g/kg IVIg dosing, but only one possibly related TEAE (elevated blood pressure of moderate severity) when treated with 1 g/kg IVIg. Another patient experienced several mild and moderate expected TEAEs under 2 g/kg IVIg but only once a mild headache during the reduced IVIg period. At the lower dose, no TEEs occurred, and there were no TEAEs leading to discontinuation of the study drug.

### Premedication

Premedication for infusions was given to 10 patients (21.3%) in the IVIg group and 4 patients (8.3%) in the placebo group. In the overall period, premedication was needed by 12 patients (12.6%) receiving IVIg. The most common types of premedication were analgesics and systemic antihistamines, each given to 6.3% of patients. Glucocorticoids were not permitted as premedication. Baseline characteristics of patients on IVIg who received premedication and those who did not were similar, suggesting that none of these factors was associated with requirement for premedication.

### Outcomes

In the first period, 3 patients (5.8%) who had received IVIg experienced 8 TEAEs leading to discontinuation of the study drug. Six of these events occurred in a single patient and were considered to be related to study drug (Table 7). The other two events (sepsis and basilar artery stenosis) were reported in one patient each and were considered to be unrelated to study drug.

**Table 7:** Possibly and probably related adverse events leading to discontinuation of study drug (overall period)

Treatment at time of event	Patient	MedDRA preferred term	Intensity	Serious	Causality	Outcome
First period IVIg	Patient 1	Muscle spasms	Severe	Yes	Probable	Recovered/resolved
		Sinus tachycardia	Moderate	No	Probable	Recovered/resolved
		Chills	Mild	No	Probable	Recovered/resolved
		Fever	Mild	No	Probable	Recovered/resolved
		Dyspnoea	Severe	Yes	Probable	Recovered/resolved
		Back pain	Moderate	No	Probable	Recovered/resolved
Extension period (all IVIg)	Patient 2	Deep vein thrombosis (TEE)	Severe	Yes	Probable	Recovered/resolved
		Pulmonary embolism (TEE)	Severe	Yes	Probable	Recovered/resolved
	Patient 3	Vertigo	Moderate	No	Possible	Recovered/resolved
		Vision blurred	Mild	No	Possible	Recovered/resolved
		Cerebrovascular accident (TEE)	Moderate	Yes	Possible	Recovered/resolved with sequelae
	Patient 4	Pulmonary embolism (TEE)	Moderate	Yes	Possible	Recovered/resolved with sequelae
	Patient 8	Musculoskeletal pain	Mild	No	Possible	Recovered/resolved
		Paraesthesia	Mild	No	Possible	Recovered/resolved
		Dizziness	Mild	No	Possible	Recovered/resolved
		Condition aggravated^a^	Mild	No	Not related	Recovered/resolved
	Patient 9	Headache	Moderate	No	Probable	Recovered/resolved
		Nausea	Moderate	No	Probable	Recovered/resolved
	Patient 10	Hypersensitivity	Mild	No	Probable	Recovered/resolved
	Patient 11	Vomiting	Mild	No	Probable	Recovered/resolved

^a^Assessed as unrelated to study drug but included for completeness of information for patient 8. *IVIg* Intravenous immunoglobulin, *MedDRA* Medical dictionary for regulatory activities, *TEE* Thromboembolic event

In the extension period, 10 patients (10.5%) who received IVIg experienced a total of 17 TEAEs leading to discontinuation of the study drug. The most common events leading to discontinuation were ‘condition aggravated’ (preferred term), which led to withdrawal of 3 patients (3.2%; 2 not related, 1 unlikely related), and PE, which led to withdrawal of 2 patients (2.1%; 1 possibly related and 1 probably related). In total, non-related TEAEs leading to discontinuation included three events of condition aggravated and one event of *Escherichia bacteraemia*. Related TEAEs leading to discontinuation are presented in Table 7.

No deaths were reported during the study.

## Discussion

Dermatomyositis is a subtype of a group of rare systemic autoimmune diseases called idiopathic inflammatory myopathy (IIM), for which there is no cure. Treatment focuses on suppressing or modulating the autoimmune response to restore muscle performance, skin, lung and other organ involvement. IVIg formulations have previously been used off-label for dermatomyositis treatment in combination with immunosuppressive therapies. Primary results from the ProDERM study have been reported separately [12] showing that IVIg is efficacious and generally safe in patients with dermatomyositis. The additional data presented herein provides evidence that IVIg treatment has a favourable safety and tolerability profile in the treatment of patients with dermatomyositis.

Of 95 patients receiving IVIg in the ProDERM study, only 8 discontinued therapy due to drug-related TEAEs. Most TEAEs were reported during or within 72 h of receiving an infusion and were mild and short lasting, with similar latency times and duration between treatment groups. There were no haemolytic transfusion reactions or deaths reported. Most patients in the study received a combination of immunosuppressive drugs and IVIg.

Previously reported safety data of IVIg treatment in dermatomyositis is limited. One randomised controlled trial, a 3-month crossover trial comparing IVIg and prednisone to placebo and prednisone in 15 refractory adult patients, reported better efficacy with IVIg as compared to placebo [22]. As in the ProDERM study, patients tolerated IVIg infusions well; however, two patients experienced severe headache with each infusion, necessitating treatment with narcotics [22]. Nevertheless, these patients still experienced a major improvement in their condition following IVIg treatment and stated that the benefit far outweighed the adverse effect [22].

In juvenile dermatomyositis, a 4-year review of nine children also reported that headaches were common after treatment with IVIg, especially after the initial treatment [23]. Headaches were mostly mild, but four of the nine children also experienced severe episodes. Two patients experienced diarrhoea, one severe nausea and one fever [23]. The authors noted considerable variability of side effects, and that lengthening the infusion to 5 days rather than 3 prevented IVIg-related side effects [23]. In a non-randomised study of IVIg in 20 adults with refractory polymyositis or dermatomyositis in combination with prednisone and cyclosporin A, overall safety results noted only minor adverse events including gastrointestinal intolerance (nausea or vomiting) [24]. A recent open-label trial of IVIg in newly diagnosed IIM patients (including nine dermatomyositis patients) also reported mild and transient flu-like symptoms, with no adverse events leading to study withdrawal [25]. Similar mild to moderate adverse reactions were also reported in another randomised study from Japan [26].

The aforementioned side effects from IVIg are similar to those observed in other diseases, where more than 80% of IVIg-associated side effects are mild and occur during or shortly after infusion [27]. A retrospective study of IVIg patients with neuromuscular disease found a similar pattern of AEs, including headache, nausea and fever; although the rates cannot be compared fairly as the patients, dosing regimens and study design differed between this and our analysis [28]. However, taken together, other published studies confirm that most of the observed TEAEs in the ProDERM study are consistent with the known safety profile of IVIg therapy.

More serious adverse reactions associated with IVIg administration include TEEs, with arterial TEEs being the most common [27]. In 2013, the US Food and Drug Administration (FDA) mandated that IVIg products include a black box warning regarding the risk of TEEs, which have been reported in 0.5–15% of patients treated with IVIg [29]. In a 10-year retrospective study assessing IVIg-related adverse events in different diseases, tolerability varied significantly between individuals and IVIg preparation [30]. For the preparation of IVIg used in this study specifically, the rate of TEEs in a study including all indications was 3 in 21,780 infusions, of which 1 was deemed possibly related to IVIg [31]. In a study of patients with neurological disorders, the rate of TEEs was 1 in 3374 infusions [32], and in patients with immune thrombocytopenia, there were no cases of TEE with 626 infusions [33]. In a recent cohort study of 458 patients with a definitive diagnosis of dermatomyositis, six of 178 patients (3.4%) who received IVIg in the preceding 4 weeks experienced venous thromboembolism (VTE) versus 16 of 280 patients who had not received IVIg. No significant difference was found between groups, suggesting that IVIg was not associated with an increase in VTE risk in these patients [34]. In the ProDERM study, patients with known history of TEE were excluded; however, results from the overall analysis showed that patients with certain risk factors for TEEs were more likely to experience TEEs than those without. Similarly, a study of data from the UK Biobank from 502,492 individuals on IVIg found that the rate of TEEs was threefold higher in patients with a history of TEE than those without [35].

Systemic inflammation associated with dermatomyositis may also increase the risk of TEEs. Systemic inflammation is postulated to modulate thrombotic responses by upregulating procoagulants, downregulating anticoagulants and suppressing fibrinolysis [36]. Indeed, there are several reports of a higher risk of TEEs in patients with dermatomyositis compared to the general population [37–39]. These reports included different variables, such as duration of disease, age and sex, although none looked specifically at treatment (including IVIg). For example, a Swedish study that used nationwide registers found 26.8 (95% confidence interval: 0.9, 52.6) venous thromboembolic events occurred in every 1,000 person-years in dermatomyositis patients (*n* = 154) versus 2.4 (0.9, 3.8) in the general population (*n* = 4,459), with a hazard ratio of 16.44 [40]. Hence, TEE monitoring in patients treated with long-term, high-dose IVIg for dermatomyositis is recommended throughout the duration of IVIg treatment as latency of TEEs is highly variable in different patients. In this study, patients who experienced TEEs had a Wells score of 0 at their last visit prior to the event. Therefore, additional risk assessments might help to prevent TEEs.

One possible way to reduce the rate of side effects is to reduce the rate of infusion [27], and this was supported by results from this analysis showing that reducing the infusion rate from 0.12 to 0.04 mL/kg/min was important in mitigating TEEs. TEE complications can also be prevented through greater vigilance in high-risk subjects, as well as the judicious use of anticoagulation therapy [27].

Besides reducing the rate of IVIg infusion, the rate of other IVIg-related TEAEs can be reduced by co-administering or pre-medicating with paracetamol, antihistamines or glucocorticoids [27, 30]. In the current study, routine prophylactic premedication was not permitted and premedication was only required for a small number of patients who had experienced two consecutive infusion-related AEs; analgesics and systemic antihistamines were most commonly administered.

Limitations of the trial include a short follow-up time of less than a year, which did not permit the capture of longer-term safety data. Also, specific subsets of dermatomyositis, including juvenile dermatomyositis, cancer-associated and amyopathic dermatomyositis, were excluded from the study, preventing our safety data from being translatable to these subgroups. The TEE risk factors highlighted in this study were identified based on the general medical history of the subjects and were not formally weighted. In addition, smoking status, a classic risk factor for TEE, was not assessed in the study.

## Conclusions

This is the first large international, randomised, placebo-controlled phase 3 trial demonstrating the safety and tolerability of IVIg as a treatment for patients with active dermatomyositis. Safety and tolerability of high-dose IVIg administration for patients with active dermatomyositis were as expected, with headache, fever and nausea being most commonly reported during or after IVIg infusion, followed by quick recovery. Patients receiving high-dose IVIg for dermatomyositis should be monitored for TEEs, and for patients with a known history of TEE, the risk/benefit of IVIg should be thoroughly discussed. In patients with multiple risk factors for TEEs, lowering the infusion rate is one of several strategies that can mitigate this risk.

### Supplementary Information

**Additional file 1: Suppl. Table 1.** Most frequently experienced related TEAEs.

## Data Availability

Access to the data underlying this paper is tightly governed by various legislative and regulatory frameworks. De-identified clinical and laboratory data and response to treatment data for the study cohort included in this study can only be made available to legitimate researchers and clinicians from medical and academic institutions, for academic and clinical research on request to Octapharma Pharmazeutika Produktionsges.m.b.H. A proposal with a detailed description of study objectives and a statistical analysis plan will be requested. The proposal will be evaluated based on European and international data protection regulations and regulations about secondary use of patient data. After approval of a proposal, de-identified data will be shared through a secure online platform upon signing a data processing agreement. The study protocol, statistical analysis plan and main results are available at https://clinicaltrials.gov/ct2/show/NCT02728752.

## Abbreviations

AEs: Adverse events
DVT: Deep vein thrombosis
FDA: Food and Drug Administration
IIM: Idiopathic inflammatory myopathy
IVIg: Intravenous immunoglobulin
MedDRA: Medical dictionary for regulatory activities
PE: Pulmonary embolism
TEAEs: Treatment-emergent adverse events
TEEs: Thromboembolic events
UK: United Kingdom
US: United States
USA: United States of America
