# Supplementary material for: Safety and tolerability of intravenous immunoglobulin in patients with active dermatomyositis: results from the randomised, placebo-controlled ProDERM study
Source: Arthritis Res Ther. 2024 Jan 17;26:27. doi: 10.1186/s13075-023-03232-2 (PMC10792872; doi:10.1186/s13075-023-03232-2)
Supplement: Supplementary file 1 — Additional file 1: Suppl. Table 1. Most frequently experienced related TEAEs. [file 13075_2023_3232_MOESM1_ESM.docx]

**Suppl. Table 1. Most frequently experienced related TEAEs**

| ***TEAE***  **N (%) [n]** | **Related TEAEs*** | | |
| --- | --- | --- | --- |
|  | **First period** | | **Overall period** |
|  | **IVIg**  **(n=52**^†^**)** | **Placebo**  **(n=48)** | **All IVIg**  **(n=95)** |
| Any related TEAE event | 30 (57.7) [113] | 11 (22.9) [38] | 62 (65.3) [282] |
| Most frequent related TEAEs (experienced by ≥5% of patients in any group), % |  |  |  |
| Headache | 19 (36.5) [46] | 3 (6.3) [7] | 40 (42.1) [92] |
| Fever | 10 (19.2) [15] | 3 (6.3) [3] | 18 (18.9) [27] |
| Nausea | 6 (11.5) [11] | 2 (4.17) [2] | 15 (15.8) [26] |
| Vomiting | 3 (5.8) [3] | 0 | 8 (8.4) [26] |
| Chills | 16 (30.8) [30] | 1 (2.08) [2] | 7 (7.4) [12] |
| Musculoskeletal pain | 3 (5.8) [3] | 0 | 7 (7.4) [7] |
| Blood pressure increased | 3 (5.8) [3] | 3 (6.3) [4] | 6 (6.3) [9] |
| Coombs test positive | 2 (3.85) [2] | 0 | 5 (5.3) [5] |
| Fatigue | 3 (5.8) [4] | 1 (2.08) [1] | 2 (2.11) [3] |

* Related adverse events included those that were deemed probably or possibly related to study drug by the investigator.

^†^ Includes 47 patients randomised to receive IVIg plus 5 patients who switched from placebo to IVIg

IVIg, intravenous immunoglobulin; N, number of patients; n, number of events; TEAE, treatment-emergent adverse event.
